# Applications of the Novel Midpalatal Piezocorticotomy Guide for MARPE Midfacial Skeletal Expansion

**DOI:** 10.3390/jcm14134728

**Published:** 2025-07-03

**Authors:** Svitlana Koval, Viktoriia Kolesnyk, Daria Chepanova

**Affiliations:** 1 DrKoval Orthodontics, Boca Raton, FL 33431, USA; info@drssk.com; 2 Department of Obstetrics, Gynecology and Reproductive Sciences, Yale School of Medicine, Yale University, New Haven, CT 06510, USA; viktoriia.kolesnyk@yale.edu

**Keywords:** MARPE, piezocorticotomy, piezoguide, midpalatal expansion, adults

## Abstract

**Background/Objectives**: MARPE expansion is known to produce maxillary skeletal expansion and cause subsequent increase in velopharyngeal, oropharyngeal, and nasal volume. While MARPE designs vary and may include combinations of traditional bands, traditional jackscrews, or milled/3D printed frameworks with other screw designs, there is no agreement on the techniques of MARPE expansion in adult patients. The aim of this case series is to describe a novel 3D-printed and 3D-designed midpalatal piezocorticotomy guide and its benefits for MARPE-assisted midfacial skeletal expansion. **Materials and Methods**: This case series showcases the results of successful MARPE expansion in adults and introduces the novel 3D-printed midpalatal piezocorticotomy guide. It compares the outcome of non-guided piezocorticotomy-assisted maxillary skeletal expansion and outlines the benefits of pre-planned 3D-guided midpalatal piezocorticotomy. **Results**: The MARPE expansion outcomes when combined with 3D-designed and 3D-printed midpalatal piezocorticotomy guides are shown to be predictable and capable of preventing asymmetric expansion along with asymmetric suture opening. The technique, in particular, allows for one to preserve the position of the nasal septum and prevents asymmetrical nasal septum dislodgement following maxillary skeletal expansion. **Conclusions**: The described novel midpalatal piezocorticotomy guide presents a significant improvement to adult midfacial techniques using MARPE expansion appliances.

## 1. Introduction

Multiple studies have demonstrated the efficacy of MARPE (Mini screw-Assisted Rapid Palatal Expander) in producing skeletal maxillary expansion in adult patients [1,2], as well as the stability [3] of these outcomes. Other studies have shown that MARPE can reduce daytime sleepiness and improve symptoms in patients with obstructive sleep apnea (OSA) [4,5].

While the effects of MARPE expansion in adults are well documented, there is a wide variety of techniques and appliance designs used to achieve skeletal maxillary expansion in this population, resulting in variable efficiency and outcomes.

A meta-analysis by Ventura et al. [6] evaluated the short- and long-term effects of MARPE on the upper airway and alar dimensions in non-growing patients. The study reported significant increases in nasal cavity width (mean difference: 2.05 mm), nasal floor width (mean difference: 2.13 mm), nasal cavity volume (mean difference: 1.24 cm^3^), and alar width (mean difference: 1.47 mm). These findings suggest that MARPE effectively expands the nasal and maxillary structures, potentially improving nasal breathing. The study also found significant reductions in nasal resistance and increases in nasal airflow following MARPE treatment, likely attributed to the expanded nasal cavity dimensions.

Adjunct techniques such as midpalatal corticotomy and piezocorticotomy have been shown to facilitate suture separation, with measurements of 3.14 mm at the premolar area and 2.06 mm at the molar area of maxillary crest-to-crest bone separation. This approach effectively addresses transverse maxillary deficiency in adult patients [7]. Although systematic reviews favor the conclusion that MARPE produces significant clinical changes compared to conventional rapid palatal expanders, surgically assisted rapid palatal expansion (SARPE), or controls, they highlight a lack of consistent methodology and outcome data [6].

Guided MARPE insertion with a 3D-planned and pre-printed temporary anchorage device (TAD) guide was described in a case report by Minervini et al. [7], which focused on the design and positioning of MARPE-supporting mini screws rather than guided piezocorticotomy for midpalatal dissection.

The aim of this case series is to describe a novel 3D-printed and 3D-designed midpalatal piezocorticotomy guide and its benefits for MARPE-assisted midfacial skeletal expansion.

## 2. Materials and Methods

All study participants have signed a written consent to participate in the research prior to commencement of the study.

### 2.1. Description of MARPE Installation Procedure

**Figure 1:**
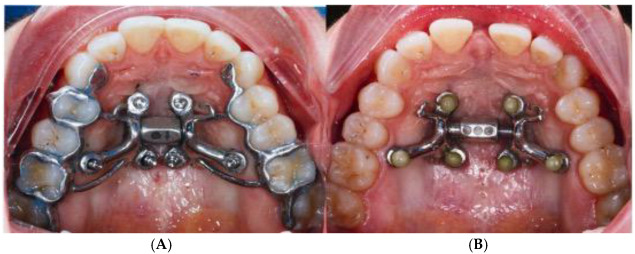
MARPE expander before and after expansion. (**A**) Guided midpalatal piezocorticotomy appointment with MARPE installation. (**B**) MARPE expansion completed and midpalatal piezocorticotomy incision healed, and the MARPE framework was adjusted with reducing the tooth-borne components.

The MARPE installation is preceded by a guide-assisted midpalatal piezocorticotomy (Piezosurgery touch, Mectron, Hilliard, OH, USA) under local anesthesia. The procedure begins with a fit check of the midpalatal piezocorticotomy guide, adjustment of the occlusal fit if needed, and the initial notch placement as specified by the guide design. The incisions are made to the depth of the palatine bone and the crests of the maxillary bones. Proprioceptive feedback and pre-measured bone depths help determine cortical plate penetration. Piezocorticotomy tips are pre-calibrated for various incision depths. One week of healing is allowed before MARPE activation begins. Activation proceeds at one turn/day for females over 8–9 weeks and at two turns/day for males until the initial midpalatal suture separation is achieved, followed by one turn/day for an additional 6–7 weeks (Figure 1).

### 2.2. Description of the Novel Piezocorticotomy Guide for Midpalatal Skeletal Expansion

The midpalatal piezocorticotomy guide is an auxiliary appliance designed to enhance midpalatal piezocorticotomy results. This technique and design, filed under US and Canadian patent applications, provides a novel method for achieving predictable midfacial bone separation.

**Figure 2:**
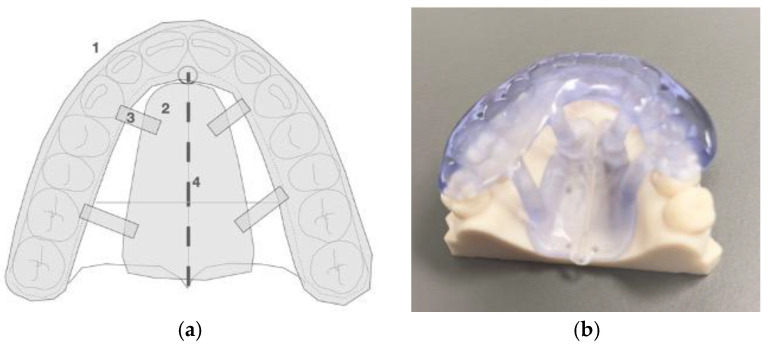
(**a**) Schematic image of the midpalatal piezoguide: 1—occlusal splint, 2—baseplate, 3—connector, and 4—piezocortical incision notches. (**b**) Piezoguide fitted on the model.

The guide is a custom-designed, 3D-printed, one-piece, unattached appliance that rests on the maxillary occlusal surfaces and palatal vault. It includes an occlusal splint, a palatal baseplate, and connectors attached to both. Printed from polymethyl methacrylate (PMMA) resin, the appliance is modifiable. The piezocorticotomy notch locations are determined via 3D planning and individualized patient anatomy (Figure 2).

The appliance design and materials used for 3D printing are modifiable to improve retention, fit, and stability. The notch locations are determined through individualized 3D planning for each patient.

When PMMA resin is used as the printing material, a thickness of 2.5 mm is recommended. The appliance functions as a temporary guide during the piezocorticotomy incision procedure.

### 2.3. Technique of the 3D Printed Guide-Assisted Midpalatal Piezocorticotomy

**Figure 3:**
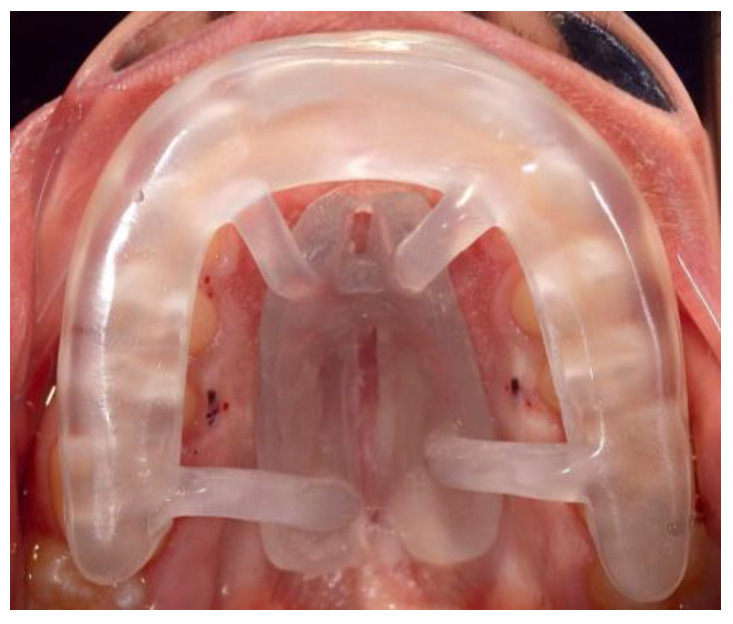
Midpalatal piezocorticotomy guide positioned in the mouth against the maxillary arch with the connectors attached to the baseplate firmly accommodated against the palatal vault. Piezocorticotomy notches are located based on the 3D planning of the location of the nasal septum.

The midpalatal piezocorticotomy technique recommended by the authors involves guide-assisted piezocorticotomy utilizing CBCT-guided planning. This technique enables bilateral separation of the maxillary crests of the palatal processes and the palatine bones (Figure 3).

**Figure 4:**
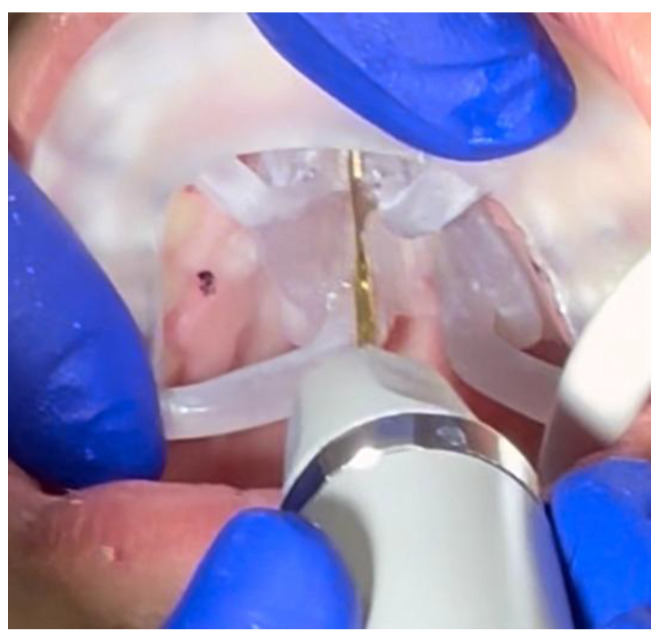
Intraoperative application and positioning of the midpalatal piezocorticotomy guide. The guide is firmly attached against the maxillary teeth due to the presence of indentations on the intaglio of the occlusal part of the guide. The clinician approaches the incision through the pre-planned notches in the baseplate of the guide.

The incision extends from the distal aspect of the incisive foramen, in the projection of the nasal septum attachment, to the most distal margin of the palatine bone at the level of the posterior nasal spine (PNS) (Figure 4).

### 2.4. Case A: Blind Midpalatal Piezocorticotomy with MARPE Expansion

**Figure 5:**
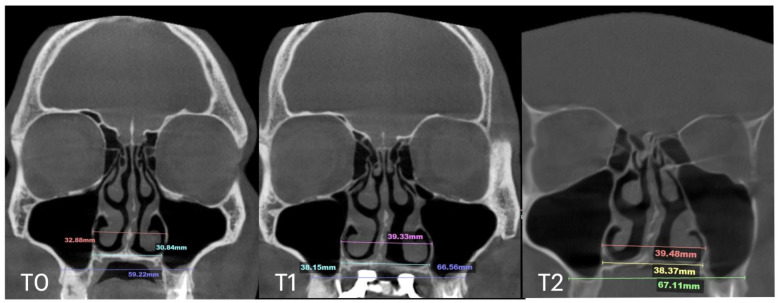
Before (**T0**) and after expansion (**T1**) with MARPE midfacial expansion without guide-assisted midpalatal piezocorticotomy, and in 12 months after expansion (**T2**). The Expansion of 10.5 mm was achieved over the 12 weeks of expansion with 1 turn/day (**T1**), complete ossification of the midpalatal suture is seen in 12 months (**T2**).

A 25-year-old male patient underwent MARPE midfacial expansion with midpalatal piezocorticotomy, opting not to use the piezocorticotomy guide. The patient presented with a skeletal Class III base, anterior open bite, and posterior open bite. The procedure was performed under local anesthesia with topical and infiltrative application in the midpalatal area. Landmarks were visually positioned along the mucosal outline of the midpalatal suture, followed by piezotome incisions (Piezosurgery touch, Mectron, Hilliard, OH, USA). Expansion followed a one-turn/day protocol over 12 weeks. The result (Figure 5) revealed unfavorable suture separation with asymmetrical nasal septum displacement and unilateral nasal airway expansion favoring the left side.

**Figure 6:**
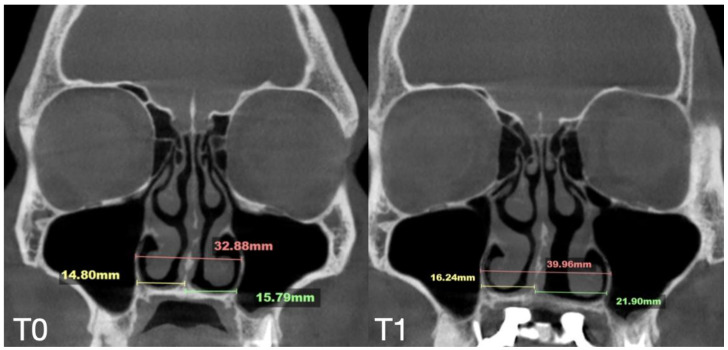
Pre- (**T0**) and post-MARPE (**T1**) expansion measurements with the blind midpalatal piezocorticotomy. (**T0**) Nasal base measurements before MARPE expansion after blind midpalatal piezocorticotomy: lateral nasal width equals 32.88 mm and the right and left septum-lateral wall widths equal 14.80 and 15.79 mm, correspondingly. (**T1**) Post-MARPE expansion measurements: lateral nasal width equals 39.96 mm and the right and left septum-lateral wall widths resulted in 16.24 and 21.90 mm, respectively.

The total expansion of 10.32 mm was distributed unevenly, with approximately 75% of the total expansion occurring in the left passage (Figure 6).

**Figure 7:**
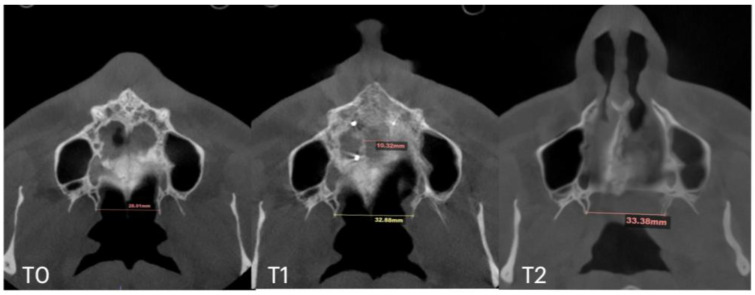
Pre- (**T0**), post-expansion (**T1**), and at the 12 months follow up (**T2**) evaluation of the midpalatal suture in axial projection. Inter-pterygoid distance was measured at all time points, pointing at a significant increase from 28.01 to 32.88 mm, which remained stable in the 12 months after expansion.

Subsequent evaluation of the suture changes was made at 12 months after expansion. Axial slices were compared to evaluate the pattern of suture separation and the characteristics of bone ossification. Figure 7 shows changes in suture presentation at T0 (before treatment), T1 (immediately after expansion), and at T2 (12 months after completion of expansion). Complete ossification is evident with no loss to inter-pterygoid distance.

### 2.5. Case B: Guide-Assisted Midpalatal Piezocorticotomy

**Figure 8:**
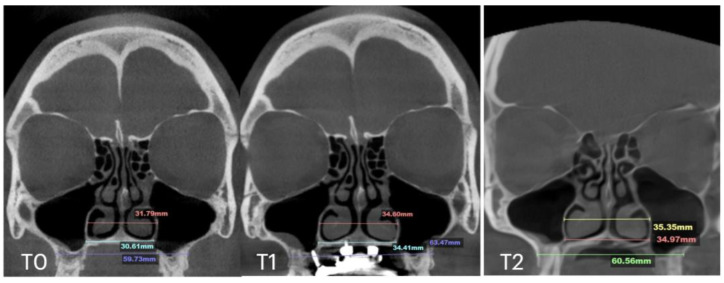
Before (**T0**) and after (**T1**) MARPE midfacial expansion with guide-assisted midpalatal piezocorticotomy, and 11 months after the completion of expansion (**T2**). The expansion of 5.7 mm was achieved over the 9 weeks of expansion with 1 turn/day. Stability of the nasal base width is evident at T1 and T2. Ossification of the suture is confirmed in coronal projection of the septum view.

A 35-year-old female underwent midfacial skeletal expansion using a custom-milled MARPE appliance. The MARPE installation was preceded by guide-assisted midpalatal piezocorticotomy under local anesthesia. Incisions reached the palatine bone and maxillary crests, with proprioceptive feedback used to confirm cortical plate penetration. After 1 week of healing, MARPE activation began at one-turn/day over 9 weeks, achieving 5.3 mm of maxillary crest-to-crest separation. Pre- and post-expansion CBCT images in the coronal projection are shown in Figure 8.

**Figure 9:**
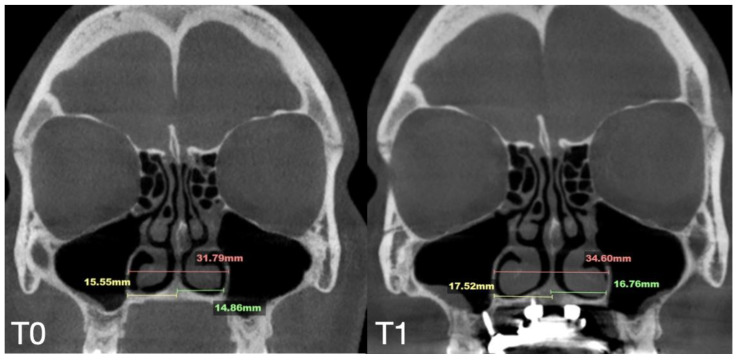
Pre- (**T0**) and post-MARPE (**T1**) expansion measurements with the blind midpalatal piezocorticotomy. (**T0**) Nasal base measurements before MARPE expansion after guided midpalatal piezocorticotomy: lateral nasal width equals 31.79 mm and the right and left septum-lateral wall widths equal 15.55 and 14.86 mm, correspondingly. (**T1**) Post-MARPE expansion measurements: lateral nasal width equals 34.60 mm and right and left septum-lateral wall widths resulted in 17.52 and 16.76 mm, respectively.

The case demonstrated a precise 5.7 mm midpalatal suture separation and a post-expansion nasal septum position centered relative to the nasal base (Figure 9).

**Figure 10:**
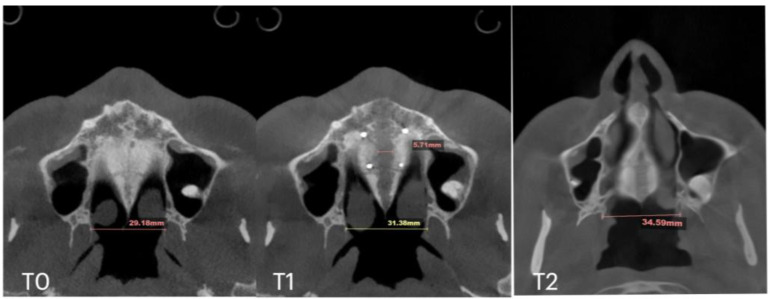
Pre- (**T0**), post-expansion (**T1**), and at the 11 months follow up (**T2**) evaluation of the midpalatal suture in axial projection. Inter-pterygoid distance was measured at all time points, pointing to a significant increase from 29.18 to 34.59 mm.

At the 11 months follow up appointment, midpalatal suture ossification was confirmed. Suture separation at ANS and PNS is visible at T1. T2 shows evidence of opaque ossified tissue along the projection of the previously radiolucent midpalatal suture separation. The inter-pterygoid distance shows stable results of expansion (Figure 10).

### 2.6. Case C: Bilateral Posterior Crossbite with Guide-Assisted Expansion

**Figure 11:**
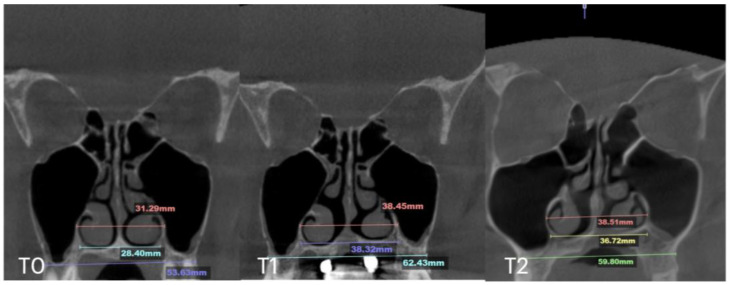
Before (**T0**) and after (**T1**) MARPE midfacial expansion with guide-assisted midpalatal piezocorticotomy, and at the 10 months follow up (**T2**). The expansion of 8.4 mm was achieved over the 10 weeks of expansion with 1 turn/day. Midpalatal suture ossification is visible, with less dense bone located below the nasal septum.

A 33-year-old male presented with an anterior open bite and bilateral posterior crossbite. He underwent MARPE expansion with guide-assisted midpalatal piezocorticotomy, performed under local anesthesia with topical and infiltrative applications. Antibiotic premedication was administered 1 h before the procedure (Figure 11).

**Figure 12:**
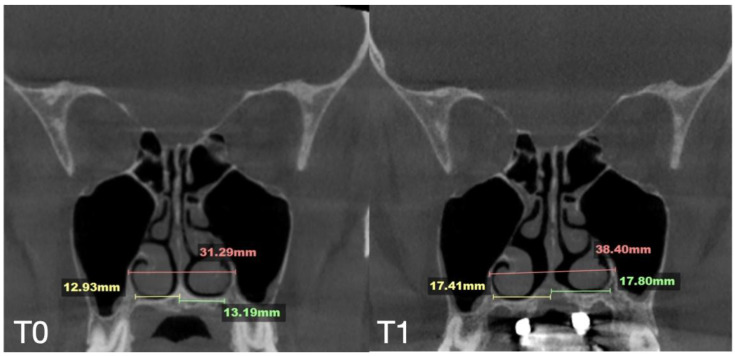
Pre- (**T0**) and post-MARPE (**T1**) expansion measurements with the blind midpalatal piezocorticotomy. (**T0**) Nasal base measurements before MARPE expansion after guided midpalatal piezocorticotomy: lateral nasal width equals 31.29 mm and the right and left septum-lateral wall widths equal 12.93 and 13.19 mm, correspondingly. (**T1**) Post-MARPE expansion measurements: lateral nasal width equals 38.40 mm and right and left septum-lateral wall widths resulted in 17.41 and 17.80 mm, respectively.

Expansion followed a one-turn/day protocol for over 10 weeks, achieving a total crest-to-crest separation of 8.47 mm. The nasal septum remained centered, and the nasal passages showed a relatively even increase in volume. The septum maintained alignment with surrounding structures, including the ethmoid cells, maxillary sinuses, and nasal cavity base (Figure 12).

### 2.7. Case D: Midfacial Asymmetry Correction with Guided Expansion

A 40-year-old female presented with maxillary roll, with the left occlusal plane positioned inferior to the right. CBCT confirmed the palatal plane was not parallel to the orbital plane.

**Figure 13:**
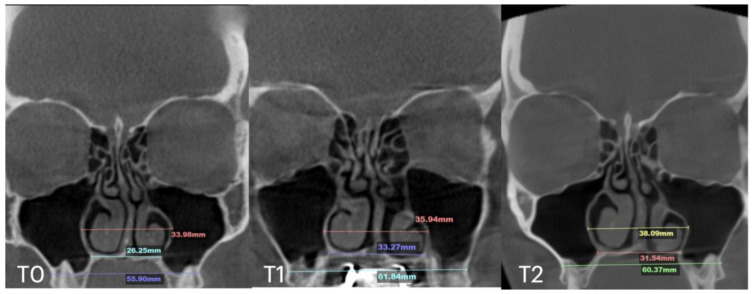
Before (**T0**) and after (**T1**) MARPE midfacial expansion with guide-assisted midpalatal piezocorticotomy, and at the 10 months follow up appointment (**T2**). The expansion of 6.2 mm was achieved over the 9 weeks of expansion with 1 turn/day. Evidence of stable nasal base width is shown at (**T1**) and (**T2**).

Midpalatal guide-assisted piezocorticotomy and MARPE insertion were performed in a single appointment, including antibiotic premedication and local anesthesia via topical and infiltrative methods. Activation began in week 2, after the initial mucosal healing period, and continued for 10 weeks (Figure 13).

**Figure 14:**
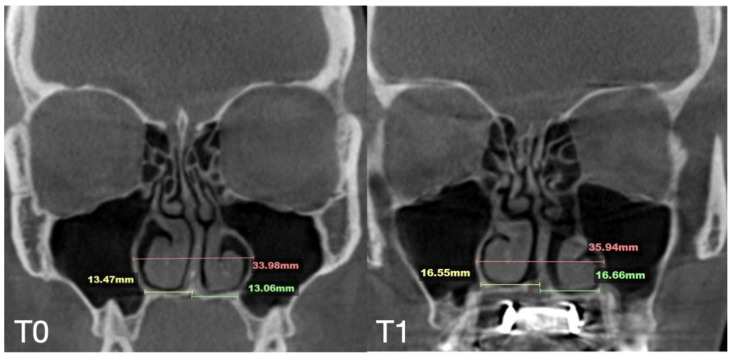
Pre- and post-MARPE expansion measurements with the blind midpalatal piezocorticotomy. (**T0**) Nasal base measurements before MARPE expansion after guided midpalatal piezocorticotomy: lateral nasal width equals 33.98 mm and the right and left septum-lateral wall widths equal 13.47 and 13.06 mm, correspondingly. (**T1**) Post-MARPE expansion measurements: lateral nasal width equals 35.94 mm and the right and left septum-lateral wall widths resulted in 16.55 and 16.66 mm, respectively.

The total expansion exceeded 6 mm, resulting in symmetrical nasal base separation and alignment parallel to the original maxillary base plane inclination (Figure 14).

**Figure 15:**
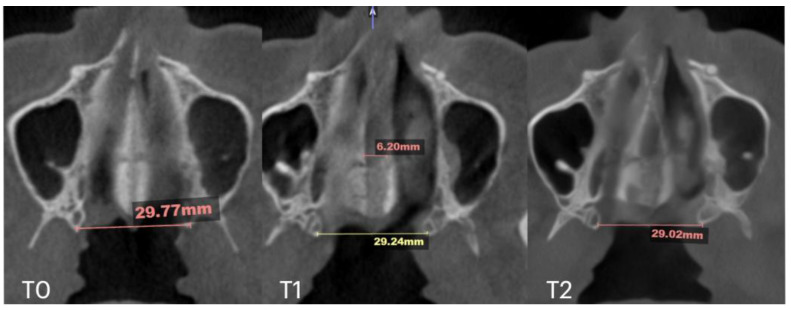
Pre- (**T0**), post-expansion (**T1**), and at the 10 months follow up (**T2**) evaluation of the midpalatal suture in axial projection. Inter-pterygoid distance was measured at all time points, pointing at relatively stable behavior in this area despite 6.2 mm of expansion and clear signs of ossification of the midpalatal suture.

Axial slices were evaluated for the amount of midpalatal separation at T1 and the stability of expansion at the level of PNS, measured as an inter-pterygoid distance (Figure 15).

Measurements of the nasal base width, nasal lateral width, maxillary base width, inter-pterygoid distances, and respective midpalatal suture crest-to-crest separation are shown in Table 1.

## 3. Discussion

The skeletal component of maxillary expansion was first described by Haas and Isaacson [8,9]. Isaacson conducted measurements using a built-in dynamometer in a fixed skeletal expander appliance and demonstrated that the forces generated by the appliance, under consistent activation regimens, ranged from 2.5 to 10 pounds. He concluded that the primary resistance to skeletal activation originated from the peri-maxillary sutures rather than the midpalatal suture itself. He also emphasized that appliance activation may vary depending on the patient’s age and midpalatal suture resistance. Isaacson described retention as highly dependent on surrounding skeletal structures rather than tooth-borne retention alone. The key to retention lies in neutralizing external forces from peri-maxillary sutures, muscles, and ligaments, which otherwise could reduce the volume of newly formed bone. The study concluded that lower force levels with low load-deflection rates are preferable for expansion mechanisms.

A study by Weissheimer et al. [10] comparing the effects of Haas and Hyrax expanders for skeletal maxillary expansion in individuals aged 9–14 supported previous findings for a V-shaped midpalatal suture separation pattern. This pattern was attributed to the resistance from the medial and lateral pterygoid plates of the sphenoid bone to the expansion-induced tipping moment. Both expander types achieved similar skeletal gains of approximately 40%, with the remaining 60% attributed to dentoalveolar changes. Notably, this study reported statistically greater skeletal expansion with the Haas expander compared to the Hyrax, contrary to earlier findings [11].

The literature lacks consensus on adult midpalatal expansion techniques, including appliance types and activation protocols. Most studies indicate beneficial changes in upper airway volume and minimum cross-sectional area [12,13,14]. A systematic review and meta-analysis by Kapetanovic et al. [1] on MARPE effects in adults found skeletal width increases ranging from 1.63 to 3.03 mm and dentoalveolar increases from 5.50 to 7.59 mm, with significant dental tipping. A study by Tang et al. [15] with similar skeletal separation demonstrated that MARPE outcomes in young adults were relatively stable. Greater stability was observed in individuals with thicker palatal bone and shallower palatal vaults [15]. Naveda et al. measured midpalatal bone density 16 months post-MARPE in young adults [16], finding that the anterior region exhibited the highest bone repair rate. Specifically, 80.95% of the midpalatal suture length was repaired in all patients, with the middle third showing the least ossification. Similar findings were reported by Ceschi et al. [17], who suggested that older patients may require longer retention periods for full ossification of the midpalatal suture. Our findings confirm a consistent and predictable pattern of midpalatal suture ossification in adult patients, irrespective of whether guided or unguided piezocorticotomy was employed. Specifically, all four patients exhibited radiographic evidence of midpalatal suture ossification within 10 to 12 months following the completion of expansion. The degree and distribution of ossification varied among the cases. Notably, at the 12-month follow up in Case A—where an expansion of 10.32 mm was achieved—a substantial amount of ossified bone was observed at the anterior nasal spine (ANS), posterior nasal spine (PNS), and at the level of the transverse palatal suture. These findings suggest that radiographically detectable bone formation typically occurs between 10 and 12 months after expansion has concluded.

Laudemann et al. reported a positive correlation between SARPE with pterygomaxillary disjunction and posterior skeletal and alveolar expansion [18]. Gherlone and coauthors [19] reported a stable maxillary advancement outcome after distraction osteogenesis in a cleft palate patient when combined with Le Fort I procedure.

To the best of our knowledge, asymmetric expansion has not been addressed in previous studies.

Recent advances in airway-focused orthodontics have shown that nasal base expansion—particularly at the levels of the anterior nasal spine (ANS), posterior nasal spine (PNS), and piriform foramen—can significantly increase airway volumes in the nasal, nasopharyngeal, oropharyngeal, and hypopharyngeal regions [14,18,20]. However, not all MARPE designs consistently achieve separation at both the ANS and PNS [18]. The present study demonstrated consistent separation of the midpalatal suture at the level of the posterior nasal spine (PNS) across all cases, further corroborated by the observed increase in inter-pterygoid distance in Cases A, B, and C. This finding is of particular significance due to its implications for the expansion of velopharyngeal airway volume and the increase in its minimal cross-sectional area. The increased inter-pterygoid distance facilitates lateral displacement of the nasopharyngeal walls, thereby contributing to an overall enlargement of the nasopharyngeal airway. Kyung et al. [21] previously described the positive effects of increased lateral pharyngeal dimensions, noting significant improvements in symptoms associated with obstructive sleep apnea (OSA). These results warrant further investigation regarding the potential application of MARPE in the treatment of OSA.

The technique described below has consistently produced midpalatal suture separation at both the ANS and PNS, along with disjunction of the pterygomaxillary sutures. These outcomes result in the forward and downward movement of the maxillary complex. Our findings consistently demonstrate per-maxillary suture separation following guided midpalatal piezocorticotomy-assisted MARPE (Microimplant-Assisted Rapid Palatal Expansion) for midfacial expansion. This process involves physiological separation of the frontomaxillary, nasomaxillary, frontonasal, zygomaticomaxillary, and pterygomaxillary sutures in adult patients. These observations suggest that guided piezocorticotomy-assisted midfacial expansion with MARPE operates via a distinct biomechanical mechanism and yields different outcomes compared to surgically assisted rapid palatal expansion (SARPE), which entails direct surgical separation of the midpalatal suture and often includes concomitant Le Fort I osteotomy to facilitate additional maxillary movement.

The current clinical case series presents a novel 3D-designed and 3D-printed midpalatal piezocorticotomy guide that enhances MARPE midfacial expansion through guided midpalatal piezocorticotomy. The pre-determined placement of piezocorticotomy notches enables precise targeting of the nasal septum base, thereby facilitating its separation from the maxillary crests. This technique promotes even and symmetrical displacement of the maxillary bones. In Cases B, C, and D, the nasal septum orientation was either preserved (B, C) or improved (D), attributed to the symmetrical expansion of the nasal base width. In contrast, Case A exhibited no observable improvement in septal orientation or morphology. Notably, all cases reported substantial enhancements in nasal breathing capacity and overall nasal airway patency.

Adult MARPE expansion—especially in males over 18 years of age—has historically been considered questionable, with alternative techniques often recommended to improve nasal airflow and address nasal breathing as a component of sleep-disordered breathing. For this age group, variations in piezocorticotomy have been suggested [22]. SARPE (surgically assisted rapid palatal expansion) is frequently combined with Le Fort I osteotomy to address maxillary size, shape, and position relative to the skull base. While SARPE combined with Le Fort I was shown to improve obstructive sleep apnea is young adults [23], MARPE has become a promising and less-invasive solution.

The technique described in this article has proven effective and efficient for midfacial expansion in adult males and females over 18, achieving maxillary crest-to-crest separation exceeding 5 mm. The benefits of using the guided midpalatal piezocorticotomy include the prevention of unilateral nasal septum displacement, asymmetric nasal cavity and volume expansion, maxillary base roll, and vertical discrepancies of the maxillary occlusal plane.

### Rationale for the Use of the Midpalatal Piezocorticotomy Guide

Unfortunately, current research lacks prospective studies comparing adult MARPE expansion with and without midpalatal piezocorticotomy. As a result, the topic remains primarily within the domain of clinical observation, debate, and discussion.

Our clinical observations indicate that when midpalatal separation—measured at the level of the maxillary crest above the first molars—exceeds 5 mm of crest-to-crest distance, the following complications are frequently observed:

- Asymmetric expansion with residual attachment of the nasal septum unilaterally to the maxillary crestal bone (Figure 5 and Figure 6);
- Diagonal fractures of the palatine bone extending from the distal margin of the palatal process of the maxillary bone unilaterally, often associated with pain and tension;
- Asymmetric displacement of the nasal base floor with unilateral downward and outward movement (Figure 5 and Figure 6);
- Downward inclination and displacement of the alveolar process on the ipsilateral side (Figure 5 and Figure 6);
- Unilateral soft tissue shift, including nasal ala and corner of the mouth displacement toward the side of unattached separation.

The midpalatal piezocorticotomy guide is designed to mitigate most of these complications. It accounts for each patient’s unique anatomy to enhance both the efficiency and symmetry of maxillary expansion. Key contributing anatomical variables include the position and symmetry of the nasal septum and variability in palatal bone thickness.

**Figure 16:**
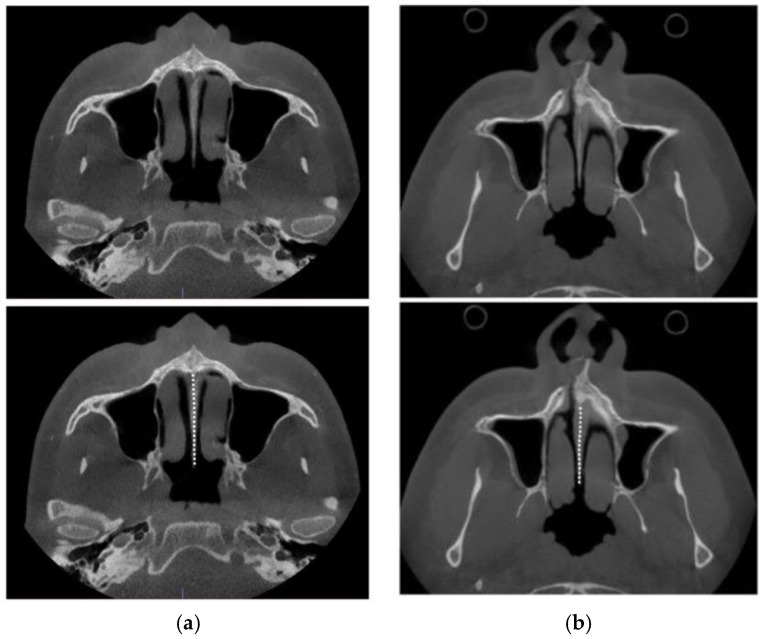

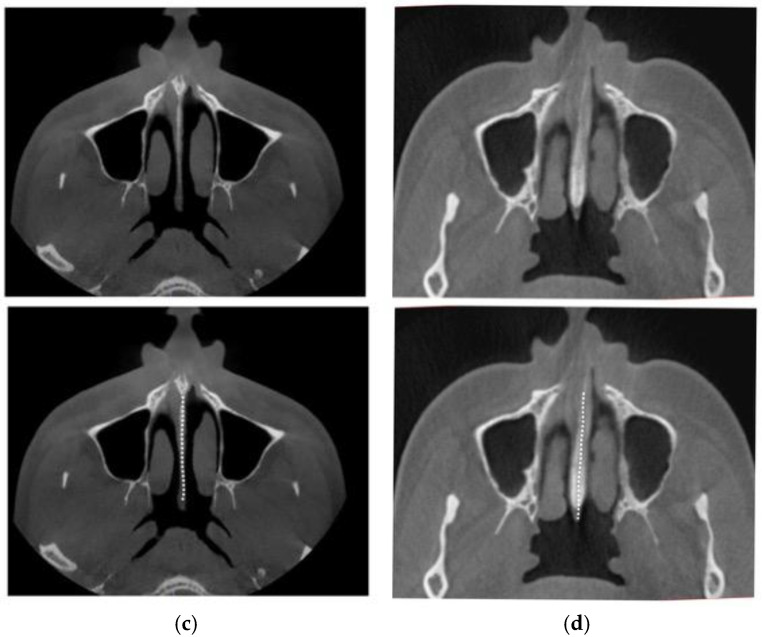
Base of the nasal septum of a 40 y.o. female (**a**), 35 y.o. male (**b**), 23 y.o. male (**c**), and 35 y.o. female (**d**) in the axial plane (before intervention).

The nasal septum’s attachment projection is best visualized in axial CBCT views of the maxillofacial region. Figure 16 illustrates variations in septal shape and attachment.

As shown in Figure 16, nasal septum variability extends beyond the coronal plane and poses additional challenges when evaluated in the axial plane. This complexity contributed to the asymmetric nasal septum displacement observed in Case A.

**Figure 17:**
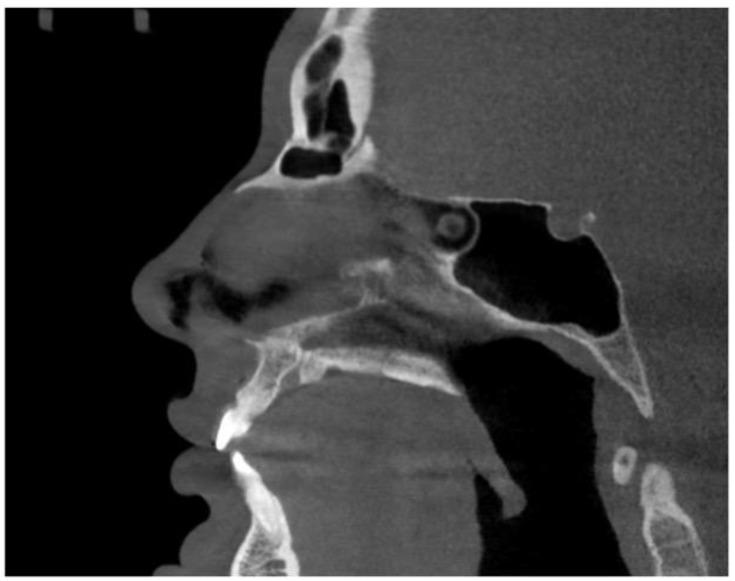
Sagittal midline view of the hard palate with the incisive canal cross-section.

The depth of the piezocorticotomy incisions involves the full thickness of the palatine bone and the maxillary crest in the corresponding regions, as shown in the sagittal view (Figure 17).

Case A, which involved midpalatal separation without guided piezocorticotomy, represents a typical outcome of blind piezocorticotomy. In our experience, such cases consistently lack expansion symmetry. Common findings include unilateral nasal septum attachment, asymmetrical lateralization of the nasal walls, uneven bone apposition on the contralateral side of the septum, vertical drop of the dental-alveolar complex, and other complications.

The potential risks associated with guided midpalatal piezocorticotomy are minimal and substantially reduced compared to those of non-guided procedures. This reduction in risk is primarily attributed to the high precision enabled by 3D software planning and 3D printing technologies. These tools allow for accurate determination of penetration depth and location, significantly decreasing the likelihood of damaging the incisive nerve and artery. Furthermore, the use of instruments with marked depths ensures precise control during the procedure.

## 4. Conclusions

The novel midpalatal piezocorticotomy guide represents a significant advancement in adult midfacial expansion using the MARPE appliance. Guided piezocorticotomy of the midpalatal suture not only helps prevent major side effects but also improves the predictability and symmetry of maxillary expansion in adult male and female patients while minimizing potential side effects and complications.

## 5. Patents

US. And Canada Patent Pending: Piezocorticotomy guide for midpalatal skeletal expansion (Application #18/919,416).

## Figures and Tables

**Table 1:** Respective measurements of the nasal base width, nasal lateral width, maxillary base width, inter-pterygoid distance, and midpalatal suture separation in mm at T0, T1, and T2.

	Nasal Base Width, mm			Maxillary Base Width, mm			Lateral Nasal Width, mm			Inter-Pterygoid Distance, mm			Midpalatal Suture Separation, mm
	T0	T1	T2	T0	T1	T2	T0	T1	T2	T0	T1	T2	T1
Case A	30.84	38.15	38.37	59.22	66.37	67.11	32.8	39.33	39.48	28.01	32.88	33.38	10.5
Case B	30.61	34.41	34.97	59.73	63.47	60.56	31.79	34.6	35.35	29.18	31.38	34.59	5.7
Case C	28.40	38.32	36.72	53.63	62.43	59.80	31.29	38.45	38.51	29.15	35.8	35.36	8.47
Case D	26.25	33.27	31.54	55.9	61.84	60.37	33.98	35.94	38.09	29.77	29.24	29.02	6.2

## Data Availability

The original contributions presented in this study are included in the article. Further inquiries can be directed to the corresponding author.

## Abbreviations

The following abbreviations are used in this manuscript:

MARPE	Mini screw-Assisted Rapid Palatal Expansion
ANS	Anterior Nasal Spine
PNS	Posterior Nasal Spine
SARPE	Surgically Assisted Rapid Palatal Expansion
OSA	Obstructive Sleep Apnea
